# Case Report: A case of functional reconstruction of second metacarpal following complex trauma with free fibula flap and silicone arthroplasty

**DOI:** 10.3389/fsurg.2025.1714330

**Published:** 2025-12-12

**Authors:** Tito Brambullo, Enrico Caporali, Federico Ricci, Vincenzo Vindigni, Franco Bassetto

**Affiliations:** Plastic Surgery Unit, Department of Neurosciences, University of Padua, Padua, Italy

**Keywords:** arthroplasty, bone transfer, flap reconstruction, free fibula, hand injuries, hybrid reconstruction, implant, metacarpal

## Abstract

Complex metacarpal fractures with segmental bone loss and joint involvement pose significant challenges for the hand trauma surgeon. When traditional techniques are inadequate, microsurgical reconstruction and joint arthroplasty may offer a viable alternative. We report the case of a 32-year-old male who sustained a high-energy injury to the right hand, resulting in a comminuted fracture of the second metacarpal head and extensive dorsal soft tissue loss. After initial management, reconstruction of the second metacarpal was performed using a free osteocutaneous fibula flap. Although the vascularized bone graft was sculpted to settle in the metacarpophalangeal joint, mobility was not preserved. A staged silicone MCP arthroplasty then was later performed to improve joint mobility. At 18 months follow-up, the patient showed good soft tissue coverage, restored grip strength and proximal interphalangeal joint motion, but improvement in MCP range of motion remained partial despite arthroplasty. This article highlights the potential and limitations of combining vascularized bone transfer with prosthetic joint replacement in high-demand patients following trauma.

## Introduction

Metacarpal fractures are a frequent occurrence in the upper limb and are commonly treated with static splinting and immobilization. However, in cases where the stumps are not properly aligned or the fracture is highly fragmented, surgical intervention is necessary. The aim of surgery is to achieve stable fixation and reduce immobilization time (1). Plate and screw fixation, Kirschner wires, and external fixation are some of the techniques available for this purpose. While closed fractures with no bone loss can be managed effectively, open fractures with loss of soft and bone tissues present a greater challenge for hand surgeons. Inadequate management of this condition can lead to complications such as pseudoarthrosis, chronic pain, deformity, and late ray loss, making it a serious concern (2).

To tackle this particular clinical situation, various treatment methods have been suggested, ranging from traditional techniques with bone graft (3) to modern microsurgical approaches. Factors such as the location of the fracture, the extent of bone loss, damage to the articular surface, and soft tissue injuries play a crucial role in determining the most appropriate treatment option. In cases where the metacarpal head and metacarpophalangeal joint are affected, it is essential to restore the articular surface or perform definitive arthrodesis to achieve a stable ray while limiting the functional recovery by immobilizing the metacarpophalangeal joint (MCP).

Two proposed solutions for addressing MCP loss issues are the osteochondral vascularized bone graft harvested from the medial femoral condyle flap or the vascularized metatarsophalangeal joint transfer from the second toe (4, 5). Both methods can replace a small amount of bone loss, but usually they are insufficient for larger defects, in which cases an interposed traditional bone graft or a second free bone flap are needed to replace the metacarpal shaft.

Some individuals may opt for double free flap reconstruction to achieve substantial functional recovery (6), whereas others might favor less invasive treatments with quicker recovery times (7). Consequently, treatment decisions should, whenever feasible, align with patients’ preferences. In our case, the patient declined microsurgical transplantation of the second metatarsal-phalangeal joint. Therefore, we proposed an alternative approach to replace the metacarpal shaft and metacarpal-phalangeal joint through a two-step procedure. This article delineates our experience utilizing a free fibula flap for shaft replacement and silicone arthroplasty for proximal MCP substitution.

## Case report

**Figure 1:**
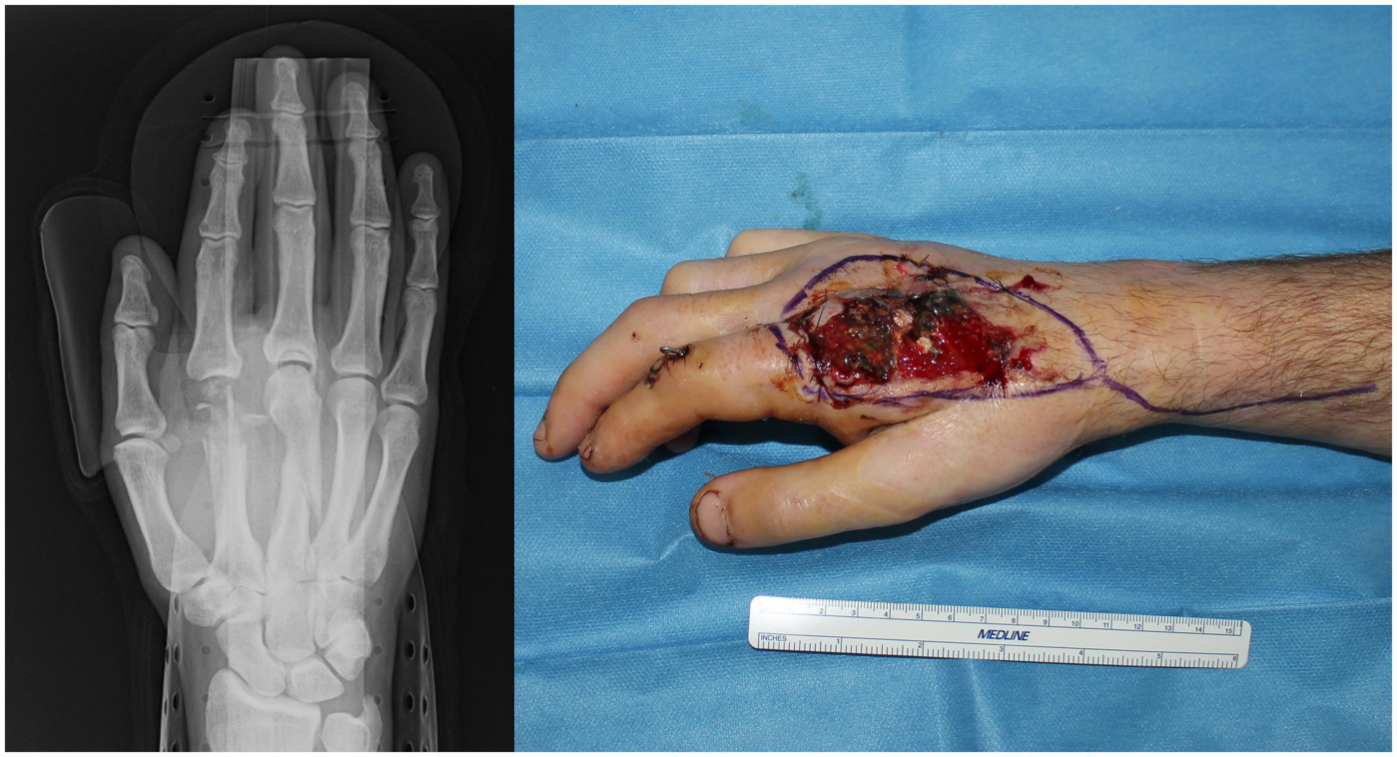
Radiographic imaging of the right hand with an open intraarticular comminuted fracture with bone loss of substance of the second metacarpal head and spiral fracture of the third metacarpal shaft (left) and preoperative picture after initial damage control.

A 32-year-old, right-handed male with no comorbidities presented to our hand trauma emergency service with a disc grinder injury of his right hand that occurred during his working activities. Physical examination demonstrated broad dorsal side skin loss of substance and complete section of the extensor digitorum tendons of the second and third fingers. The vascular injury was confined to the dorsal veins and there was an area of numbness that corresponded to the dorsal skin of the first phalanx. Radiographs revealed a spiral fracture of the third metacarpal and comminuted fracture of the second metacarpal head with significant bone loss (Figure 1). The base of the first phalanx was intact, while the radial ligament and part of the articular capsule resulted absent. Immediate temporary K-wire fixation of fractures and extensor tendons repair were performed, together with accurate debridement of necrotic tissues. Negative pressure wound therapy (ActiV.A.C. therapy unit, Solventum corporation, Maplewood, MN), and broad-spectrum intravenous antibiotics were initiated.

**Figure 2:**
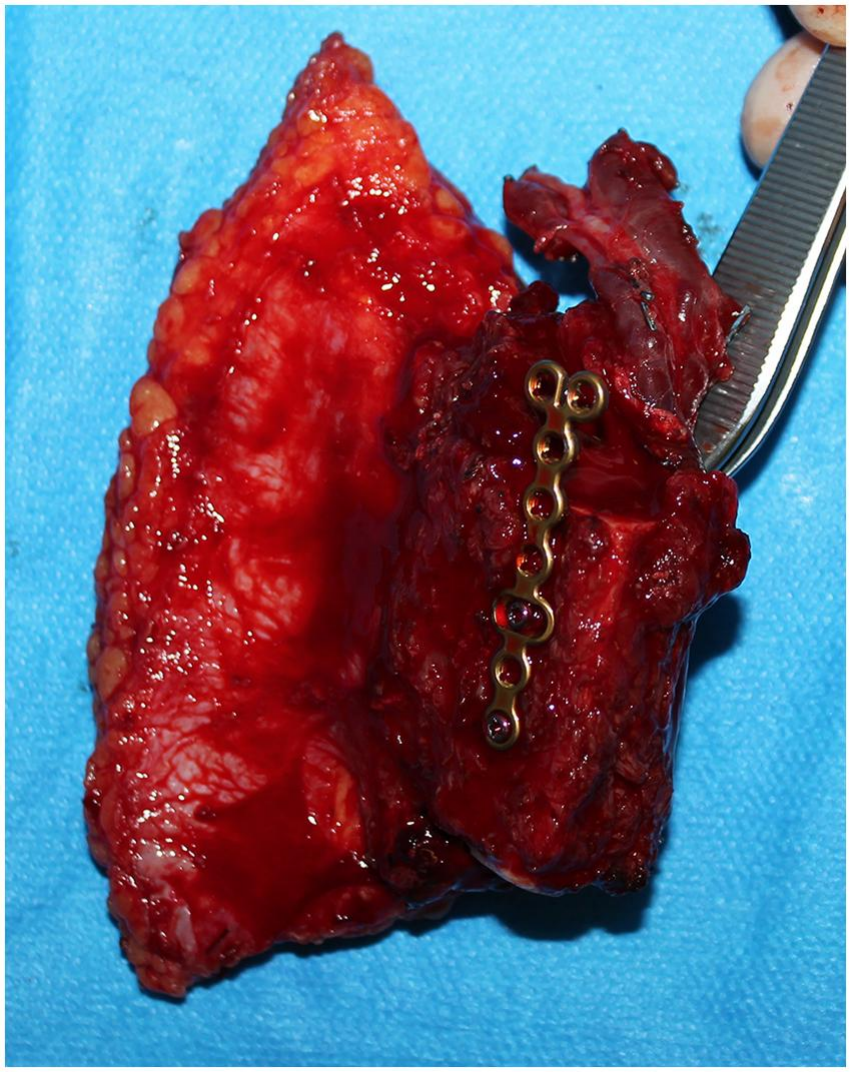
Intra operative picture of the osteo-cutaneous free fibula flap. Plate and screws were secured to the bone prior to flap detachment.

Following the initial assessment, the patient was subjected to preoperative computed tomography angiography and Doppler ultrasonography to rule out any vascular variant (i.e., peronea arteria magna) and create a map of the appropriate options and cutaneous perforators of the leg. Subsequently, the reconstructive alternatives were discussed with the patient, highlighting the advantages and disadvantages of each. The patient declined the possibility of vascularized metatarsophalangeal joint transfer from the foot and medial femoral condyle flap. As a result, ten days after the injury, bone and soft tissue reconstruction was performed using osteoseptocutaneous free fibula transfer. The flap was harvested from the right leg, with the skin paddle measuring 12 × 6 cm, tailored to the defect following soft tissue debridement. The attached bone segment measured 5 cm in length. The flap received its blood supply from two direct septal perforators (Figure 2).

The bone was stabilized proximally to the second metacarpal base using a plate and screws. End-to-side anastomosis of the peroneal artery to the radial artery and end-to-end anastomosis of the two venae comitantes to the dorsal forearm veins were performed. Additionally, the third metacarpal fracture was secured with screws. Replacement of the MCP joint was postponed due to the elevated risk of contamination and the potential for dorsal dislocation, which was attributed to the suboptimal condition of the capsule and extensor apparatus. The joint capsule and the extensor tendons were repaired.

The patient began rehabilitation the day after the surgery with the guidance of hand therapists.

During subsequent months, the second finger exhibited inward angulation and progressive joint stiffness. Radiographic examination disclosed osteophyte formation. The second-stage surgery involved osteotomies with bone burr, MCP release, and correction of the varus deformity of the first phalanx using a palmaris longus tendon graft to replace the radial collateral ligament. The rehabilitation program was then repeated. At the nine-month follow-up, there was maintenance of the correct digital axis angulation and good bone integration; however, the patient experienced high-grade MCP joint stiffness, while the proximal interphalangeal articulation (PIP) maintained a full range of motion.

Although the patient experienced no limitations in daily activities, an improvement in MCP range of motion was necessary; thus, a proposal for MCP joint reconstruction with silicone implant arthroplasty was made.

**Figure 3:**
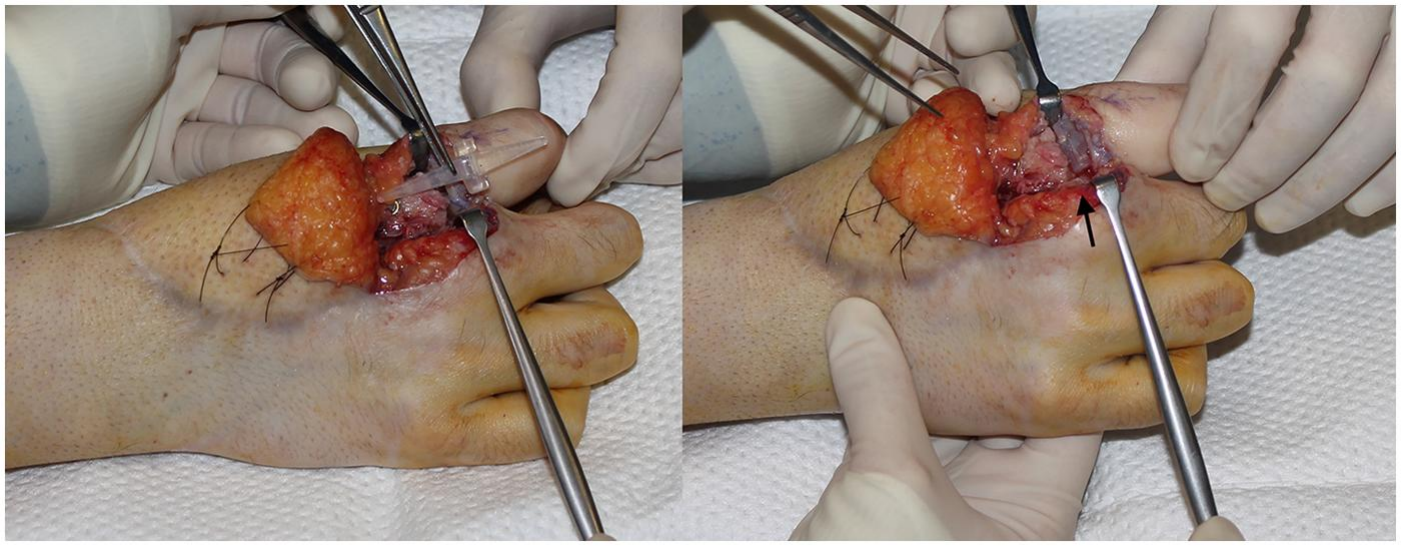
Intra operative pictures revealing inset of the swanson silicone implant (black arrow) between the fibular vascularized bone graft and the second proximal phalanx.

Bores were drilled in the medullary cavity of the fibular graft and at the base of P1 to insert a Swanson-type silicone articular implant (Wright Medical Technology, Arlington, TN) (Figure 3). The MCP capsule was secured, and an intraoperative stability check was performed to prevent implant extrusion or dislocation.

Passive physical therapy was initiated as early as two weeks post-surgery, with active therapy commencing four weeks after the procedure. A restriction on weight-bearing activities was enforced for six weeks following the operation. During outpatient follow-up, there was no evidence of wound dehiscence or implant extrusion. Despite the patient's full compliance with the rehabilitation protocol, only a minimal enhancement in metacarpophalangeal (MP) joint mobility was noted, with an approximate increase of 0–3 degrees. Even with increased exercise intensity, no significant improvement was observed. Conversely, the patient gradually resumed daily activities and reported the ability to grasp objects with full strength, without appreciable limitations, expressing overall satisfaction with the outcome.

**Figure 4:**
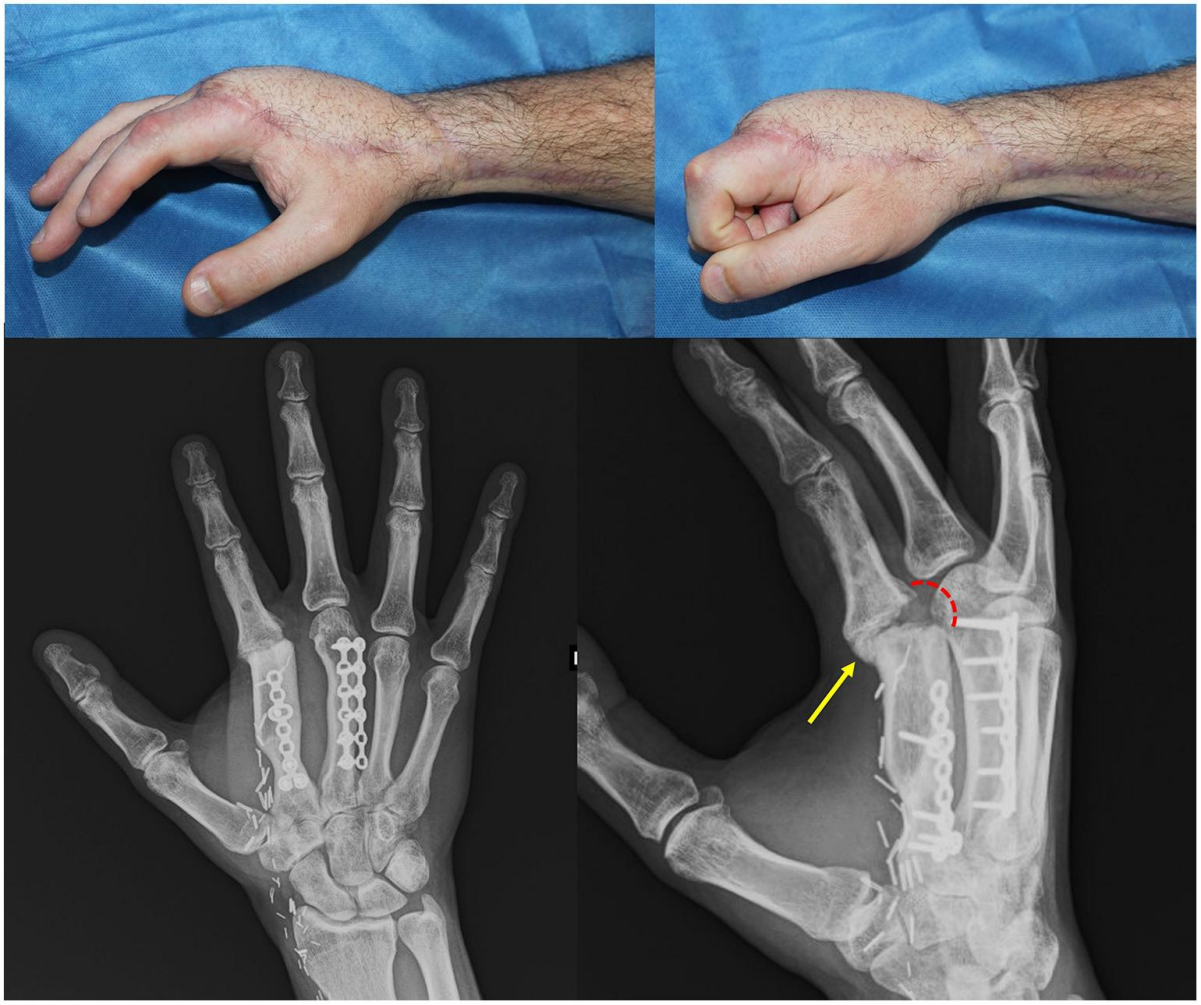
Follow-up at 18 months. Above left and right, post operative appearance exhibits very limited MCP joint mobility restoration with PIP joint full range of motion. Below left, radiographic imaging shows second finger few degrees radial deviation. Below right, volar bone spurs growth potentially interfering with joint mobility (yellow arrow), whereas intra articular space is maintained dorsally by the silicone implant (red dotted line).

The power and precise pinching were comparable to those of the contralateral side, and the overall appearance of the hand was good (Figure 4).

## Discussion

Digit reconstruction methods vary broadly, trauma-related factors, such as the nature and location of the fracture, the extent of bone loss, and the extent of soft tissue damage, as well as patient-related factors, require careful consideration as well as the surgeon's experience in microsurgery. The aim of this study was to recover the functionality of the second finger.

Among the microsurgical options, a vascularized metatarsal transfer with a matched metatarsophalangeal joint, providing a “like-with-like” reconstruction (8), was initially proposed as the preferred choice for the patient. In order to restore the MCP joint, the procedure would have involved the resection of the first phalanx's proximal articular surface. However, the donor site for the foot would have required the interposition of a bone graft or ray amputation (9).

The use of an osteochondral flap from the medial or lateral femoral condyles for metacarpal head reconstruction represents an alternative solution for addressing complex defects of the metacarpophalangeal (MCP) joint (10–12). Both techniques involve harvesting a portion of the cartilage and underlying bone from the non-weight-bearing area of the knee joint and transplanting it to the damaged metacarpal head. The primary advantage of this approach is its ability to provide a ready-made articular surface that closely mimics the natural structure and function of an original joint. Another potential advantage of femoral condyle flaps in complex traumas involving more than one metacarpal bone is the possibility of harvesting a chimeric flap based on a common origin of distant vascular branches nourishing a separate segment of cortical and cancellous bone, including a skin perforator, allowing two separate vascularized bone grafts and a skin paddle for defect coverage (13, 14). However, the success of this procedure hinges on several critical factors. The surgeon must ensure proper bone size matching between the donor and recipient areas and achieve secure fixation of the flap to the remaining metacarpal bone. Additionally, the integration of the transplanted tissue with the surrounding soft tissues, including ligaments and tendons, is crucial for maintaining joint stability and function. The size of distant vascular branches of the condyle may be insufficient. In such cases, separate free flaps should be considered for skin coverage and bone replacement. Despite their potential benefits, the long-term outcomes of these techniques remain somewhat unpredictable due to variables such as graft survival, potential donor site morbidity, and the body's response to the transplanted tissue. As a result, careful patient selection, meticulous surgical technique, and comprehensive post-operative management are essential to optimize the chances of a successful reconstruction.

Non-microsurgical techniques, such as the two-step procedure described by Masquelet (3), were also taken into account.

The patient declined these two options due to concerns regarding donor-site morbidity. Therefore, we opted for an osteocutaneous free fibula flap because of its good resistance, straight longitudinal axis, diameter, and shape, which make it suitable for reconstructing metacarpal bones (15). Moreover, the reduced thickness and pliability of the skin paddle provide optimal results in reproducing dorsal hand skin (16). After discussing the poor condition of the second finger extensor apparatus and MCP capsule with the patient, we decided to perform metacarpal reconstruction with only the peroneal bone flap, shaping the distal end to allow a minimum joint with the phalanx articular surface, without interposing an implant given the risk of dorsal dislocation. The results of the autologous reconstruction were acceptable, as the second ray was stable, grip was restored, interphalangeal joint mobility was regained, and the MCP was blocked, but not painful.

Despite this, the patient was concerned about MCP joint stiffness and asked for further improvement in the MCP range of motion.

As metacarpal soft tissue healing seemed to be definitive and stable at this time, synthetic arthroplasty was deemed advantageous, and we opted for a Swanson^TM^-type silicone implant, a monocomponent prosthesis with a dorsal flexible hinge. The combination of silicone arthroplasty and fibula osteoplasty has already been described in the literature, but almost exclusively in the setting of oncologic surgery. Athanasian et al. (17) described two cases of giant cell tumors (GCT) of the distal metacarpal treated with resection and reconstruction using a non-vascularized fibular bone graft and Swanson^TM^ arthroplasty. Manfrini et al. (18) reported a similar case of GCT of fourth metacarpal treated likewise.

Jones et al. (19) performed reconstruction of the entire fourth metacarpal and metacarpophalangeal joint using a fibular osteocutaneous free flap and silicone arthroplasty after radical oncologic resection of a giant cell tumor; however, unlike our case, they carried out joint prosthesis implantation and flap insets in a single surgical stage. In the setting of trauma surgery, Driscoll et al. (20) utilized silicone arthroplasty and fibular allograft to treat a gunshot wound in a young man in 2023. In our case, we opted for a chimeric flap with a vascularized bone graft and skin paddle because of the high risk of infection and the need for soft tissue coverage. In addition to greater resistance to infections, vascularized bone grafts show better bone integration and superior resistance to biomechanical forces compared to non-vascularized grafts (10). These factors led us to prefer delaying the arthroplasty to a later stage, as well as the need to wait for stable MCP capsule healing and evaluate the residual limitations of the new MCP joint.

The results indicated an improvement in the MCP range of motion that was approximately 0°, suggesting that the overall success of the procedure was only partially satisfactory. An x-ray scan scheduled 1 year after the arthroplasty procedure revealed that the silicone implant allowed to maintain a certain articular space dorsally, while on the volar side important formation of new bone spurs emerged. This may be due to suboptimal silicone implant positioning and mismatch between the size of the peroneal graft and the proximal phalanx. Together with the young age of the patient, these factors may have contributed to the bone spur growth, which can interfere with joint motion.

At this stage, potential solutions for the residual joint stiffness — including a vascularized metatarsophalangeal joint graft, a joint allograft, or less invasive options such as repeat arthrolysis — were thoroughly discussed with the patient. However, all these options were ultimately declined.

## Conclusions

The free osteocutaneous fibular flap provided good results in traumatic metacarpal head defects with soft tissue loss. In young manual workers with high functional demands, it can be associated with silicone arthroplasty to restore full metacarpophalangeal joint mobility.

To the best of our knowledge, this is the first case of free vascularized fibula graft and silicone arthroplasty following hand trauma reported in the literature. Even if MCP range of motion could not be restored, our findings offer new perspectives for complex digital reconstruction.

## Data Availability

The original contributions presented in the study are included in the article/Supplementary Material, further inquiries can be directed to the corresponding author.
